# FunARTS, the Fungal bioActive compound Resistant Target Seeker, an exploration engine for target-directed genome mining in fungi

**DOI:** 10.1093/nar/gkad386

**Published:** 2023-05-19

**Authors:** Turgut Mesut Yılmaz, Mehmet Direnç Mungan, Aileen Berasategui, Nadine Ziemert

**Affiliations:** Translational Genome Mining for Natural Products, Interfaculty Institute of Microbiology and Infection Medicine Tübingen (IMIT), Interfaculty Institute for Biomedical Informatics (IBMI), University of Tübingen, Auf der Morgenstelle 28, 72076, Tübingen, Germany; Translational Genome Mining for Natural Products, Interfaculty Institute of Microbiology and Infection Medicine Tübingen (IMIT), Interfaculty Institute for Biomedical Informatics (IBMI), University of Tübingen, Auf der Morgenstelle 28, 72076, Tübingen, Germany; University of Tübingen, Cluster of Excellence ‘Controlling Microbes to Fight Infections’, Auf der Morgenstelle 28, Tübingen 72076, Germany; Translational Genome Mining for Natural Products, Interfaculty Institute of Microbiology and Infection Medicine Tübingen (IMIT), Interfaculty Institute for Biomedical Informatics (IBMI), University of Tübingen, Auf der Morgenstelle 28, 72076, Tübingen, Germany; German Center for Infection Research (DZIF), Partner Site Tübingen, Tübingen, Germany

## Abstract

There is an urgent need to diversify the pipeline for discovering novel natural products due to the increase in multi-drug resistant infections. Like bacteria, fungi also produce secondary metabolites that have potent bioactivity and rich chemical diversity. To avoid self-toxicity, fungi encode resistance genes which are often present within the biosynthetic gene clusters (BGCs) of the corresponding bioactive compounds. Recent advances in genome mining tools have enabled the detection and prediction of BGCs responsible for the biosynthesis of secondary metabolites. The main challenge now is to prioritize the most promising BGCs that produce bioactive compounds with novel modes of action. With target-directed genome mining methods, it is possible to predict the mode of action of a compound encoded in an uncharacterized BGC based on the presence of resistant target genes. Here, we introduce the ‘fungal bioactive compound resistant target seeker’ (FunARTS) available at https://funarts.ziemertlab.com. This is a specific and efficient mining tool for the identification of fungal bioactive compounds with interesting and novel targets. FunARTS rapidly links housekeeping and known resistance genes to BGC proximity and duplication events, allowing for automated, target-directed mining of fungal genomes. Additionally, FunARTS generates gene cluster networking by comparing the similarity of BGCs from multi-genomes.

## Graphical Abstract

**Graphical Abstract:**
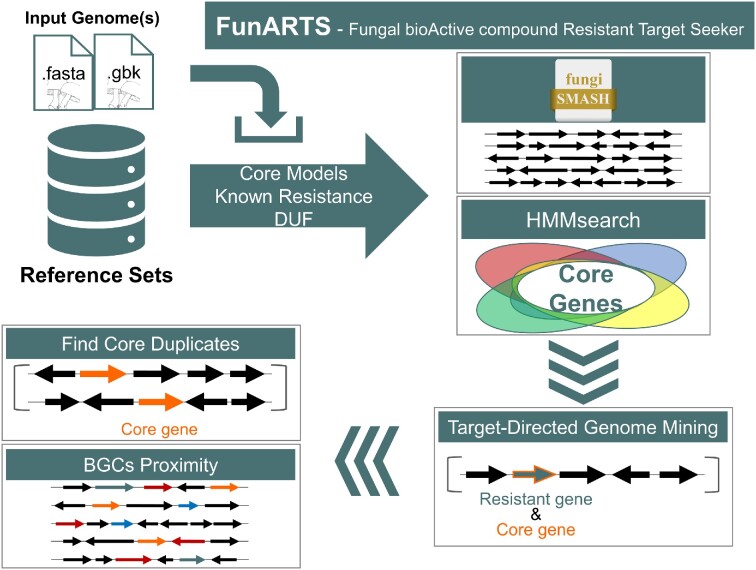

## INTRODUCTION

Antibiotic resistance is continually increasing and becoming a global problem (1). Considering that almost 700 000 people die yearly worldwide due to infections by multi-drug resistant bacteria, novel antibiotics are urgently needed (2). Most antibiotics as well as other therapeutics, such as antifungal, anticancer, or immune-inhibitory compounds are obtained as secondary metabolites by bacteria and fungi (3). Additionally, it is known that fungal species can produce a lot of bioactive compounds, such as proteasome inhibitors (4), herbicides (5) and antifungals (6). However, in the latest survey of fungal genome sequences, only a small fraction of the fungal biosynthetic space, approximately 3%, was associated with compounds (7). This highlights the vast potential of fungi as a source of new bioactive compounds with novel modes of action and underlines the need to accordingly prioritize and characterize fungal genomes.

In traditional drug discovery methods, screening of natural products and activity-based studies have played a key role. Recently, drug discovery efforts have been reinvigorated by modern bioinformatics tools and methods (8). Genome-based methods, such as genome mining, revealed silent or uncharacterized biosynthetic gene clusters (BGCs) that encode bioactive compounds (9,10). Genome mining tools and databases are steadily improved, and both allow efficient, automated, and fast mining of the thousands of available bacterial and fungal genomes. FungiSMASH (11), DeepBGC fungal (12) and TOUCAN (13) are some of the most widely accepted and used tools for fungal BGC detection. Likewise, the antiSMASH database (14), MIBiG (15), the Atlas of Biosynthetic Gene Clusters (IMG-ABC) (16), and the Mycocosm Database (17) store many thousands of well-known and predicted fungal BGCs encoding potential natural products. However, when Kelleher *et al.* studied over 1000 fungal genomes to predict BGCs and their families, they predicted 36 399 BGCs and reported 12 067 gene cluster families (GCFs). They identified 2026 BGCs in these families, which may correspond to metabolite products (7). Considering that over 1 million fungal species were estimated (18), the major challenge now is the prioritization of the most promising BGCs encoding bioactive compounds for wet lab experiments.

Recently, it has been observed that target-directed genome mining allows prioritizing bioactive compounds with potential as antibiotics or antifungals. This approach is based on the insight that antimicrobial producers must be resistant to their own products to avoid suicide (3). Often, these resistant genes are co-localized within the BGC. Various resistance mechanisms have been described, including one that involves modification of the target, which may be an essential protein. In this scenario, the BGC encodes a resistant variant of the target (19). This knowledge enables predicting the mode of action of a compound, even if a chemical structure is unknown, through the detection of a duplicated and resistant homolog of an essential gene within a BGC (20). Moore *et al.* screened orphan BGCs for a duplicated copy of fatty acid synthase genes that were found to be co-localized within orphan BGCs, thereby identifying a fatty-acid synthase inhibitor (21). Likewise, in the attempt to discover a novel proteasome inhibitor, Wang *et al.* searched for uncharacterized BGCs in *Aspergillus nidulans* and prioritized the ones containing a duplicated version of a proteasome subunit within the BGC using a target-directed approach. They eventually identified an uncharacterized and inactive BGC and managed to activate it by replacing each of its native promoters, leading to the production of the tripeptide fellutamide B (4). Another example was discovered by the Tang group (5). In their study, the researchers focused on dihydroxyacid dehydratase (DHAD). They suggested that a fungal gene cluster that produces a DHAD inhibitor may have an extra copy of DHAD for self-resistance. Through genome mining, the authors found three genes that create the tricyclic terpenoid aspterric acid, which is co-clustered with a duplicated DHAD called *astD*. This study also enabled to understand *astD* resistance to aspterric acid at the molecular level.

In 2017, we introduced the ‘Antibiotic Resistant Target Seeker’ (ARTS) (22,23). ARTS allows for the specific and efficient mining of bacterial genomes for the discovery of antibiotics with interesting and novel targets, and it is widely used by academics as well as industrial groups. Here, we present the ‘fungal bioactive compound resistant target seeker’ (FunARTS) to respond to the increasing demand for a similar tool for the analysis of fungal genomes. We developed a fungal version of our antibiotic-resistant target seeker as a user-friendly web tool that automates target-directed genome mining in all fungal taxa. The aims of FunARTS are to (i) automate the process of target-directed (also called resistance-guided) genome mining in fungi, (ii) screen for potential novel bioactive compound targets, and (iii) prioritize putative secondary metabolite gene clusters for their subsequent characterization.

## MATERIALS AND METHODS

### Workflow

**Figure 1.**
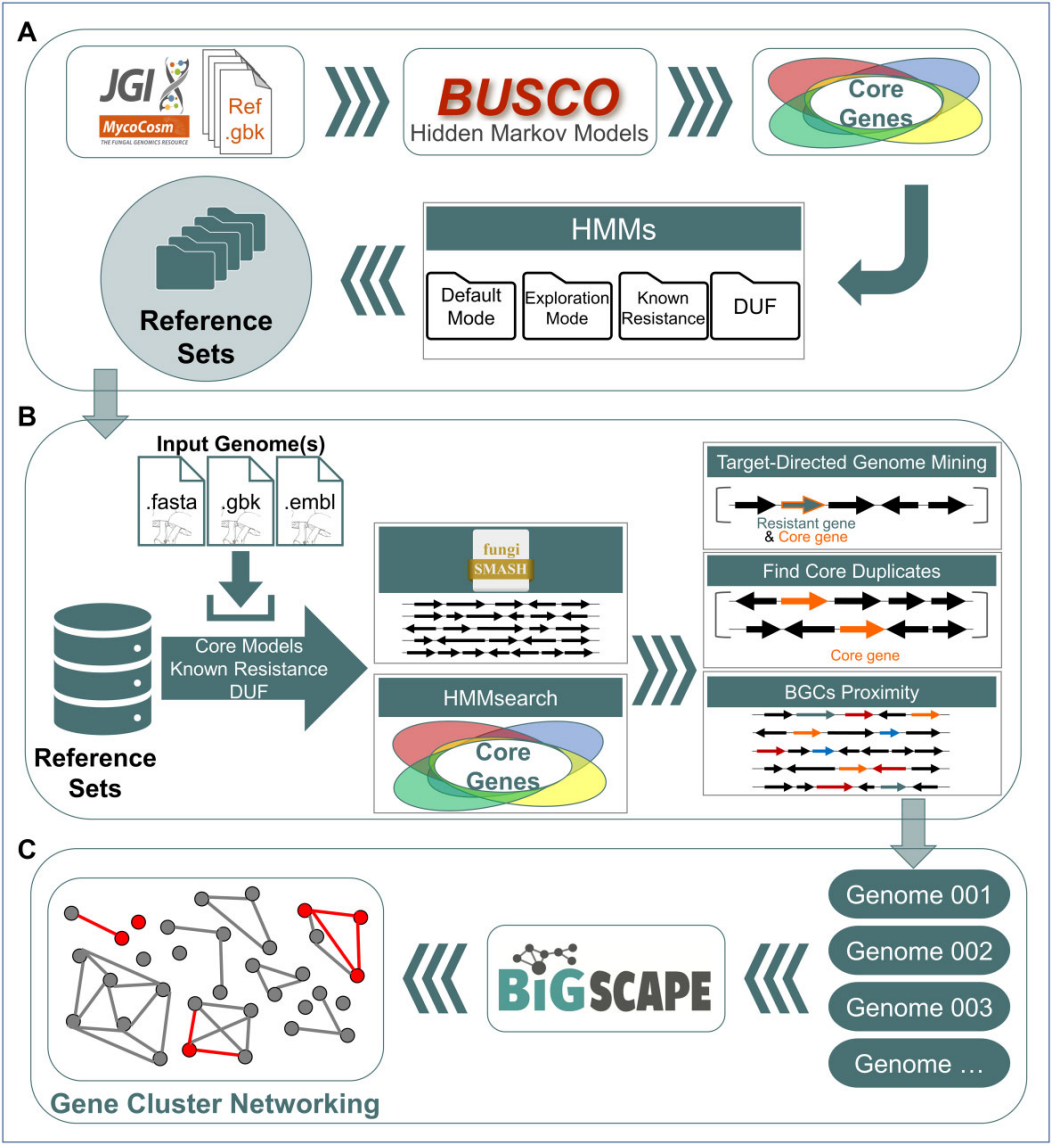
Illustration of the FunARTS pipeline. (**A**) Basic steps for reference set construction. Housekeeping core genes, duplication thresholds, and additional statistics are identified for each reference genome. The Hidden Markov Models are appended to reference sets. (**B**) The workflow of FunARTS screening. Input genome(s) are scanned to identify Biosynthetic Gene Clusters (BGCs) known resistance factors, and core genes using reference sets. FunARTS screening criteria are then applied. Duplications, BGC prioritization, and novel target discovery are integrated into the interactive output. (**C**) Comparing the results of multi-genome analysis. The BiG-SCAPE clustering algorithm is applied to all determined BGCs.

The FunARTS workflow is illustrated briefly in Figure 1. The pipeline involves these automated steps: First, users upload FASTA, Genbank, or EMBL files of fungal genomes or BGCs as input. Additionally, it is also possible to provide antiSMASH job ids. In the next step, BGCs are predicted using the antiSMASH fungal version (11). Then, FunARTS identifies essential housekeeping genes and their duplications. Essential housekeeping (core) genes within the genome are determined using Hidden Markov Models (HMMs) from the BUSCO dataset (24) by HMMER3 (25). Meanwhile, duplication thresholds are specified for each core gene model according to the reference set. Subsequently, FunARTS screens whether duplicated essential genes are located within a BGC. Additionally, it can also determine the presence of known resistance factors and domains of unknown function. Finally, interactive output tables are generated for easy exploration of the results. When multiple genomes are analyzed, the BiG-SCAPE (26) algorithm is employed to group BGCs into gene cluster families (GCFs) for easy comparison of results.

### Reference set and core gene detection

To identify reference core genes 1793 complete genomes representing 9 different phyla were collected from the MycoCosm database (Supplementary Table S1) (17). Core genes were identified using the BUSCO datasets (24) and OrthoDB v10 (27), which is one of the largest databases of orthologs with functional annotations (Supplementary Figure S1). Core genes were classified based on their function using the OrthoDB database for each reference set. As some of the core genes might be more likely associated with biosynthesis than resistance and lead to false positive results, FunARTS offers two search modes for the detection of core genes. The ‘default mode’ assists in lowering false positives and filters unlikely targets by removing regulatory, transport, and biosynthetic functions associated with known BGCs. The ‘exploration mode’ is an alternative for searching more prospective targets and skips the filtering step.

### Screening criteria

#### BGC proximity

First, fungiSMASH is used to detect BGCs. Then, BGC proximity hits are calculated by finding core and resistance gene locations that intersect with BGC boundaries determined by fungiSMASH. These hits are summarized in the interactive output tables.

#### Gene duplication

Since resistant target genes can usually be found as a second copy of the non-resistant ‘normal’ housekeeping target gene, duplication of a housekeeping gene can be an additional indicator for resistant targets (28). To identify duplication events, the median and standard deviation for the orthologs of each essential core gene was calculated and compared to the number in the query genome. All essential genes greater than these thresholds are presented in the duplication section on the result page.

### Analysis options

Several input options provide some flexibility and allow to select or deselect certain analysis options. By default, Ascomycota is selected as the reference set with all criteria and cut-offs. In addition, four other sets are available for analysis. It is also possible to search for known resistance factors and Domain of Unknown Functions (DUF). The ‘exploration mode’ option provides an extended search capability, whereas the 'Advanced' section allows users to upload their custom core genes and resistance HMM models for more customized searches.

### Output and interactive summary tables

Outputs are generated separately for single and multi-genome results in different interactive navigations. These include various sections according to the following criteria: core genes, resistance models, gene duplications, BGC proximity, and BGC networking. Users can prioritise quickly relevant properties and their combinations by searching and sorting in dynamic tables in the different sections. Additionally, expandable rows can be used for each item to investigate more detailed information. Sequence IDs are generated by FunARTS, and the location information provided in the output table is the most reliable and feasible method to match the sequences in the input file. All summary tables can be exported from the 'Export' section and saved for further analysis. FunARTS tables are provided as tsv files, and antiSMASH and BiG-SCAPE results can be visualized in a browser by opening the html-file.

### Interactive navigation for single results

The summary section depicts the number of models and screening criteria hits. Key information such as duplications, BGC proximity, and resistance models are given in the core gene table. Furthermore, functions, average selection pressure (d*N*/d*S*) values and affinity with reference organisms (ubiquity) of the essential gene are provided in the table (Supplementary Methods). The proximity table visualizes the BGC table with localized hits such as core genes or DUF. Cluster IDs ensure a link to view the original fungiSMASH results. If available, more information about the core gene model can be found in the hit table by expanding a row.

### Interactive navigation for multiple results

In the first part of the multiple results page, users can find summaries of each run and also click the links in the ‘Organism’ column to navigate through the results for each genome. The data presentation style of multiple results is similar to that of individual results. One exception is the ‘Core Genes’ table which shows the frequency (from ‘0.0’ to ‘1.0’) of hits found in the total input number rather than presence or absence. All clustered BGCs from antiSMASH results are analyzed to determine BGC similarity using the BiG-SCAPE (26) algorithm. During this analysis, the BiG-SCAPE algorithm generates sequence similarity networks of BGCs and classifies them into gene cluster families (GCFs). With an option to access the interactive big scape results page, it is possible to visualize the BGC networks of the clusters.

## RESULTS

Here we introduce the FunARTS web server, which now allows the analysis of fungal phyla, thus facilitating BGCs prioritization and discovery of bioactive compounds with interesting modes of action. To the best of our knowledge, FunARTS is the only open-source web tool that incorporates and automates the expanded, target-directed genome mining pipeline. Moreover, FunARTS offers a more user-friendly, rapid, and effective analysis compared to resistance-guided mining methods. Likewise, interactive tables and visual outputs allow uncomplicated exploration, faster interpretation, and export of all data.

### Reference set and core gene analysis

To enable accurate and efficient identification of resistance and core genes in fungal genomes, phylum-specific reference sets were generated where essential genes were identified using Hidden Markov Models. This analysis was performed using the BUSCO software. The core genes were filtered for ‘Secondary metabolites biosynthesis, transport, and catabolism’ functions by terms found in the clusters of orthologous groups (COGs) to reduce the number of false-positive targets (Supplementary Table S1). However, a second search mode, called ‘exploration mode’, is available as an option to search all the core genes in the reference beside ‘default mode’. Nonetheless, it was observed that genes with functions such as unclassified and translation, ribosomal structure, and biogenesis were the most abundant in the functional classification of each reference set (Supplementary Figure S1). Median values of all Nei-Gojobori pairwise dN/dS calculations, a gene's frequency of occurrence as a single copy, and reference ubiquity were logged to the model metadata (Supplementary Figure S2).

### Positive controls

To validate the accuracy and sensitivity of FunARTS, we analyzed most of the available fungal genomes that contained well-known examples of resistant target genes. We identified some of them following a thorough literature review. To increase the number of known positive controls, we next searched the MIBiG database for natural product BGCs with resistant target genes. From the characterized BGCs in the MIBiG dataset, corresponding genomes were downloaded and analyzed with FunARTs. However, no whole genome data are available for the squalestatin BGC. In that case only the BGC was analyzed for core genes within the BGC (Table 1). Overall, we were able to find 13 known fungal BGCs containing resistant targets. FunARTS identified at least one hit in all but four cases. In these four instances, the resistant target genes were not detected since the BUSCO database does not include these as essential genes (methionine aminopeptidase, HMG-CoA reductase, F1-ATPase ß-subunit, and ß-1,3-d-glucan synthase FKS1 subunit), showing that the database for essential genes needs to be expanded in the future. In conclusion, with few exceptions, FunARTS identified resistance genes associated with BGCs in nearly all fungal sequence data tested.

**Table 1.** Positive examples of genomes with known self-resistance mechanisms which were analyzed with the FunARTS default mode. D: duplication, B: BGC proximity, R: resistance model. Rows in bold indicate some genomes containing BGCs from the MIBiG database with known self-resistance mechanisms. The star indicates that funARTS was ran on a single BGC file

Product	Resistance gene	Organism	FunARTS hits	Criteria hits (D and B)	BGCs (total, core hit, res hit)	Genes (core, total)
Fellutamide B	Proteasome ß6-subunit (4)	*Aspergillus nidulans* FGSC A4	D, B, R	8	55, 30, 3	1515, 10 597
Mycophenolic acid	Inosine 5’-monophosphate dehydrogenase (29)	*Penicillium brevicompactum*	D, B	10	56, 34, 2	1420, 9468
Cyclosporin A	Cyclophilin (30)	*Tolypocladium inflatum* NRRL 8044	Core	5	42, 22, 4	1479, 9479
Cladosporin	Lys-tRNA synthetase (31)	*Cladosporium cladosporioides* TYU	D	3	25, 14, 1	1296, 10 784
Fumagillin	Methionine aminopeptidase (32)	*Aspergillus fumigatus* Af293	No Hit	3	37, 14, 2	1557, 9630
Lovastatin	HMG-CoA reductase (33)	*Aspergillus terreus* ATCC 20542	No Hit	12	74, 43, 2	1558, 10 509
Citreoviridin	F1-ATPase ß-subunit (34)	*Aspergillus terreus* NIH2624	No Hit	13	68, 40, 2	1490, 10 414
Echinocandin	ß-1,3-D-glucan synthase FKS1 subunit (35)	*Pezicula radicicola* NRRL 12192	No Hit	5	63, 18, 1	1029, 12 133
**Cytochalasin E**	**Angiogenesis (36)**	***Aspergillus clavatus* NRRL 1**	**Core**	**5**	**46, 17, 2**	**1562, 9230**
**Atpenin B**	**Succinate Dehydrogenase (37)**	***Penicillium oxalicum* 114–2**	**Core**	**5**	**46, 25, 2**	**1564, 9980**
**Harzianopyridone**	**Succinate Dehydrogenase (37)**	***Trichoderma harzianum* T6776**	**B**	**6**	**56, 15, 0**	**1551, 12 156**
**Squalestatin S1**	**Squalene Synthase (38)**	*Phoma sp*. MF5453*	**B**	**0**	**1, 1, 0**	**2, 22**
**Depudecin**	**Histone deacetylase (HDAC) (39)**	***Alternaria brassicicola* ATCC 96836**	**Core**	**0**	**16, 4, 0**	**1190, 9961**

## DISCUSSION

With FunARTS, we automated target-directed genome mining methods for the fungal kingdom in a single workflow. Thereby, we enabled the search for potential targets of novel bioactive compounds and the prioritization of putative secondary metabolite gene clusters for their characterization. Previous tools have attempted the prioritization of fungal BGCs for their characterization based on a resistance gene approach. The FRIGG (Fungal Resistance Gene-directed Genome mining) pipeline using Python scripts, was tested on 51 *Aspergillus* and *Penicillium* genomes leading to the identification of 72 unique resistance gene families within BGCs (40). While FRIGGs successfully allowed for the prioritization of BGCs for their subsequent characterization, it requires the user to acquire extensive bioinformatic skills. In comparison to FRIGG, the reference set of FunARTS contains all 51 genomes available in FRIGG and more. They also have common structures, such as putative resistance genes and candidate proteins. Both methods obtained similar outcomes for the mycophenolic acid cluster and the fellutamide B cluster. However, FunARTS is the only easy-to-use web tool available at the moment.

Besides the classic resistance-guided screening of known resistance models, additional criteria and tools have been included in FunARTS to assist in a rapid exploration of potential new targets. Although these criteria alone do not unequivocally represent resistance, they can highlight the enormous potential of specific fungal genomes as sources of promising bioactive compounds. Additionally, the interactive output page allows users to filter high numbers of positive hits quickly and easily. Moreover, multiple genomes can be analyzed, compared, and exported in a user-friendly way. Similarly, the BiG-SCAPE tool ensures that all detected BGCs are examined for similarity to each other.

FunARTS aims to survey a wide scope of potential genes as drug targets while minimizing manual inspection by using dynamic outputs and multiple screening criteria for more confident target predictions. However, since high numbers of false positive hits are still possible, it is incumbent on the user to examine potential hits with provided metadata, contextual framing, and expert knowledge. Some of the FunARTS hits might be more likely involved in biosynthesis and not associated with resistance, it is currently not possible to automatically distinguish if genes are more likely involved in biosynthesis or resistance. We suggest focusing primarily on the proximity criterium and checking subsequently if the core gene present within the BGC is also duplicated in order to decrease false positive hits.

In conclusion, FunARTS is a web server that allows BGC detection and novel target screening. In the future, we will work on adding new features such as the prediction of horizontal gene transfer (HGT) and phylogenetic screening. We also plan to update reference sets and existing models, such as known resistance factors. In this way, we aim to diversify the results of FunARTS by offering a wider range of applications. We have designed FunARTS to be open to new developments and to work with alternative models customized by users. Consequently, we hope to provide a more comprehensive approach to resistance-based genome mining methods in fungi to expedite the discovery of bioactive compounds.

## DATA AVAILABILITY

FunARTS is available at https://funarts.ziemertlab.com.

## Supplementary Material

gkad386_Supplemental_File
