# Supplementary material for: FunARTS, the Fungal bioActive compound Resistant Target Seeker, an exploration engine for target-directed genome mining in fungi
Source: Nucleic Acids Res. 2023 May 19;51(W1):W191–7. doi: 10.1093/nar/gkad386 (PMC10320164; doi:10.1093/nar/gkad386)
Supplement: gkad386_Supplemental_File [file gkad386_supplemental_file.pdf]

SUPPLEMENTARY DATA

Supplementary Table S1

Genome count, Hidden Markov Model, and core gene distributions for each reference set

Reference Sets	Genome Counts	Number of BUSCO markers	Amount of Core Genes in Default Mode	Amount of Core Genes in Exploration Mode
Ascomycota	1069	1706	1566	1706
Basidiomycota	553	1764	1657	1764
Microsporidia	9	600	583	600
Mucoromycota	99	1614	1520	1614
Others	63	758	703	758
**TOTAL**	**1793**	**6442**	**6029**	**6442**
Others: Blastocladiomycota, Chytridiomycota, Cryptomycota, Olpidiomycota, Zoopagomycota				

Supplementary Figure S1

Distribution of core genes detected from all reference sets according to NCBI Clusters of Orthologous Genes (COGs) functions.

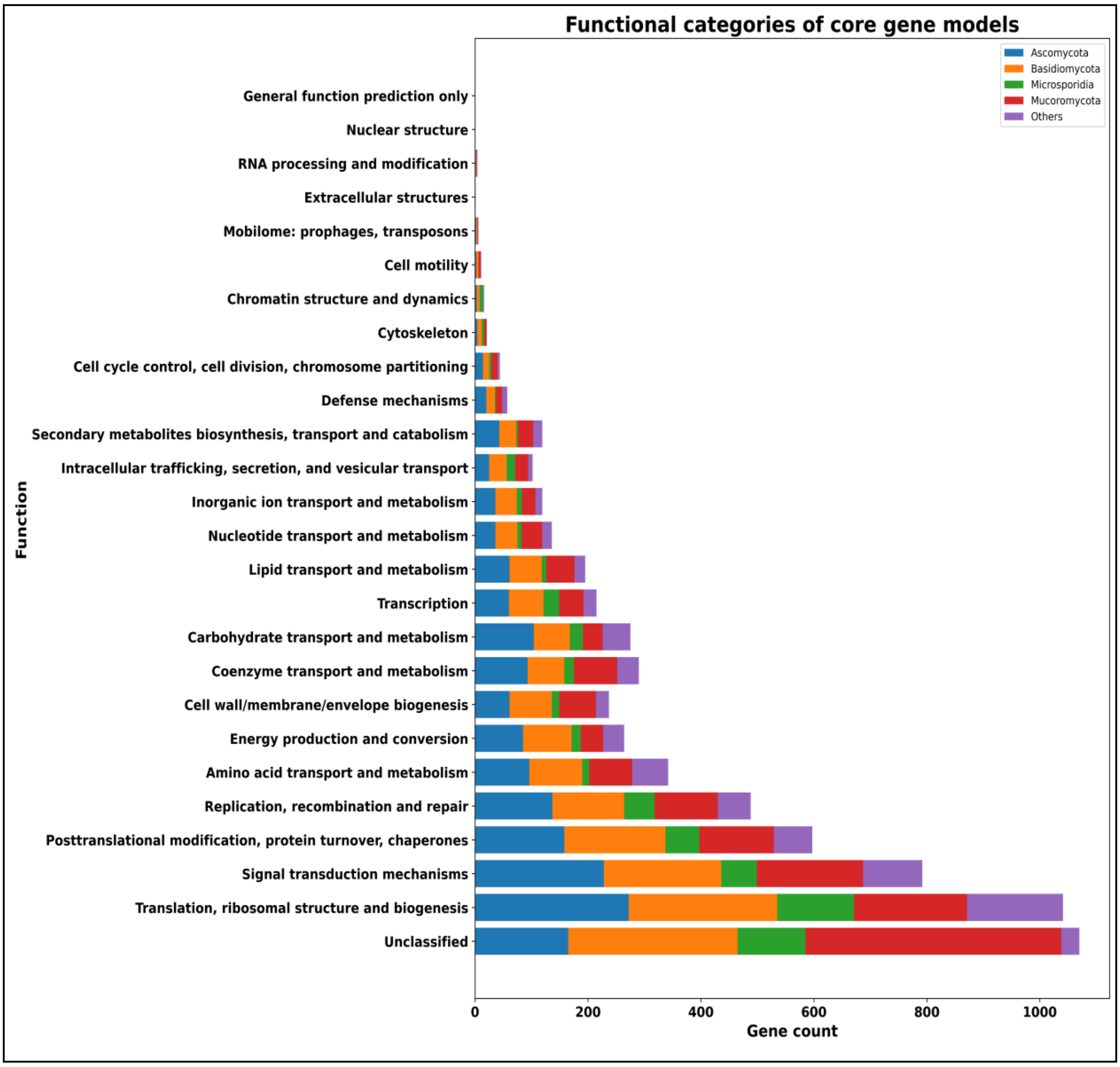

Supplementary Figure S2

Histograms of some statistical measurements. (a) The median results of the dN/dS calculations for each of the aligned core genes. (b) By examining the presence of the reference in all genomes, reference ubiquity by species is determined. A gene is counted if the majority of the creatures in a genus possess it, and these counts are displayed in relation to the total number of genera in the set. (Ratio: count with gene / total count of set) (c) The single copy ratio displays the number of instances where a gene is only detected once. The number of genomes with one copy is divided by the total number of genomes with one or more copies to obtain this ratio. (Ratio: gene count =1 / gene count >=1)

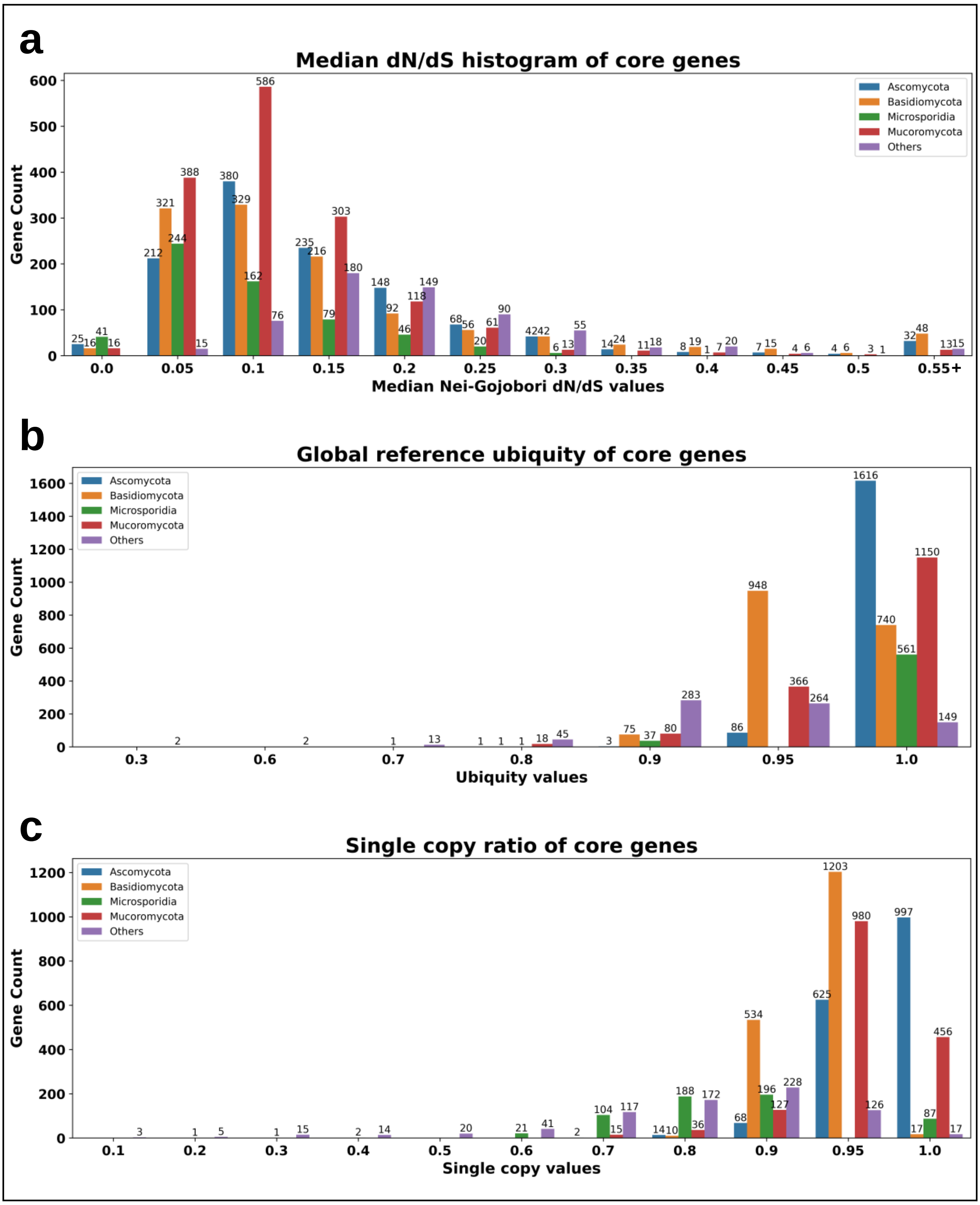

SUPPLEMENTAL METHODS

Nei-Gojobori dN/dS values

The associated multi-record FASTA files were generated by extracting all of the identified core gene sequences. Each core gene protein and codon alignment were performed using the MAFTT (1) and PAL2NAL (2) tools, respectively. Then, TrimalAl (3) was used to create trimmed copies of each aligned codon, with a preference for its most probability-optimized "automated1" setting. Each tree was constructed using RAxML (4) with 100 bootstrap replicates and GTRGAMMAI model selection. The PAML.yn00 (5) module was used to calculate the values of the pairwise selections (dN/dS) for each alignment. All of the Nei-Gojobori dN/dS median values were recorded in the model data file with other static information such as ubiquity and single copy values.

FunARTS pipeline parameters

To generate reference sets, BUSCO v5.4.3 was used to identify core genes in fungal genomes along with the cut-off values (scores and lengths) contained in BUSCO (6). Protein was selected for the "--mode" option, and specific BUSCO datasets (*_odb10) were used for each phylum for the "--lineage-dataset" option.

During funARTS analysis, AntiSMASH is run with the "--taxon fungi" and the "--minimal" parameters to detect gene clusters (7). When AntiSMASH can't find any genes and it gives an error or runs on FASTA inputs, the "--genefindig-tool" parameter is active, and it is used with glimmerHMM. To search core gene Hiden Markow Models against the input genome, the hmmsearch program of the HMMER tool is used (8). The score cut-off values provided by BUSCO are applied for the threshold value by default. The options "--noali" and "--notextw" are used to reduce the output volume and avoid limiting the length of the lines in the output, respectively. If analyzing multiple genomes, BiG-SCAPE (Biosynthetic Gene Similarity Clustering and Prospecting Engine) software is used with the "--mix" parameter, and all Biosynthetic Gene Clusters (BGCs) combine into a single network file (9).

REFERENCES

1. Katoh, K. and Standley, D.M. (2013) MAFFT multiple sequence alignment software version 7: improvements in performance and usability. *Mol Biol Evol*, **30**, 772-780.
2. Suyama, M., Torrents, D. and Bork, P. (2006) PAL2NAL: robust conversion of protein sequence alignments into the corresponding codon alignments. *Nucleic Acids Res*, **34**, W609-612.
3. Capella-Gutierrez, S., Silla-Martinez, J.M. and Gabaldon, T. (2009) trimAl: a tool for automated alignment trimming in large-scale phylogenetic analyses. *Bioinformatics*, **25**, 1972-1973.
4. Stamatakis, A. (2014) RAxML version 8: a tool for phylogenetic analysis and post-analysis of large phylogenies. *Bioinformatics*, **30**, 1312-1313.

5. Yang, Z. (2007) PAML 4: phylogenetic analysis by maximum likelihood. *Mol Biol Evol*, **24**, 1586-1591.
6. Manni, M., Berkeley, M.R., Seppey, M., Simao, F.A. and Zdobnov, E.M. (2021) BUSCO Update: Novel and Streamlined Workflows along with Broader and Deeper Phylogenetic Coverage for Scoring of Eukaryotic, Prokaryotic, and Viral Genomes. *Mol Biol Evol*, **38**, 4647-4654.
7. Blin, K., Shaw, S., Kloosterman, A.M., Charlop-Powers, Z., van Wezel, G.P., Medema, M.H. and Weber, T. (2021) antiSMASH 6.0: improving cluster detection and comparison capabilities. *Nucleic Acids Res*, **49**, W29-W35.
8. Potter, S.C., Luciani, A., Eddy, S.R., Park, Y., Lopez, R. and Finn, R.D. (2018) HMMER web server: 2018 update. *Nucleic Acids Res*, **46**, W200-W204.
9. Navarro-Munoz, J.C., Selem-Mojica, N., Mullowney, M.W., Kautsar, S.A., Tryon, J.H., Parkinson, E.I., De Los Santos, E.L.C., Yeong, M., Cruz-Morales, P., Abubucker, S. *et al.* (2020) A computational framework to explore large-scale biosynthetic diversity. *Nat Chem Biol*, **16**, 60-68.
